# Subsurface pressure profiling: a novel mathematical paradigm for computing colony pressures on substrate during fungal infections

**DOI:** 10.1038/srep12928

**Published:** 2015-08-11

**Authors:** Subir Patra, Sourav Banerjee, Gabriel Terejanu, Anindya Chanda

**Affiliations:** 1 Integrated Material Assessment and Predictive Simulation Laboratory, Department of Mechanical Engineering, University of South Carolina, Columbia, SC 29208, USA; 2 Department of Computer Science and Engineering, University of South Carolina, Columbia, SC 29208, USA; 3 Department of Environmental Health Sciences, Arnold School of Public Health, University of South Carolina, Columbia, SC 29208, USA

## Abstract

Colony expansion is an essential feature of fungal infections. Although mechanisms that regulate hyphal forces on the substrate during expansion have been reported previously, there is a critical need of a methodology that can compute the pressure profiles exerted by fungi on substrates during expansion; this will facilitate the validation of therapeutic efficacy of novel antifungals. Here, we introduce an analytical decoding method based on Biot’s incremental stress model, which was used to map the pressure distribution from an expanding mycelium of a popular plant pathogen, *Aspergillus parasiticus*. Using our recently developed Quantitative acoustic contrast tomography (Q-ACT) we detected that the mycelial growth on the solid agar created multiple surface and subsurface wrinkles with varying wavelengths across the depth of substrate that were computable with acousto-ultrasonic waves between 50 MHz–175 MHz. We derive here the fundamental correlation between these wrinkle wavelengths and the pressure distribution on the colony subsurface. Using our correlation we show that *A. parasiticus* can exert pressure as high as 300 KPa on the surface of a standard agar growth medium. The study provides a novel mathematical foundation for quantifying fungal pressures on substrate during hyphal invasions under normal and pathophysiological growth conditions.

With changes in global climate, fungal pathogens are a growing global threat to human health, agricultural sustainability, and economy^1^,^2^,^3^,^4^,^5^,^6^,^7^. Contamination of crops with mycotoxins upon infections by plant pathogens result in an annual loss of $1.3–2.5 billions only to the United States alone^8^. In addition, fungal infections in humans have significantly increased globally with the increasing immunocompromised world population over the last two decades^9^. Children and the elderly, as well as individuals undergoing organ transplantation or other major surgery, or who are suffering from AIDS are at high risk of developing life-threatening fungal infections from common human pathogens such as *Candida albicans*, *Aspergillus fumigatus*, and *Cryptococcus neoformans*^10^,^11^. It is estimated that the total direct cost for US healthcare to treat these invasive fungal infections is $2.6 billion annually with an average per patient cost of approximately $31,000^12^,^13^. Hence, there is a new drive for discovery of new antifungal molecules, which will need reliable and robust quantitative tools for determination of therapeutic efficacy. Such tools should be able to accurately compare fungal expansion and potency of hyphal penetration in a host under pathophysiological and therapeutic conditions.

To address the critical need for quantitative tools for measuring fungal invasion, we have recently developed a methodology for 3D tomography of a growing fungal colony^14^. We have already demonstrated in *Aspergillus parasiticus,* an aflatoxin producing plant pathogen, that our method which we call Quantitative Acosutic Contrast Tomography (Q-ACT), can provide physical strength profiles (viscoelastic parameters) as well as hyphal architecture at multiple scales in a growing colony^14^. In this study we have expanded the functionality of Q-ACT by investigating the physical changes that occur within the substrate beneath and around the fungal colony. It is already established that fungal invasions during infection are associated with enormous turgor pressure^15^ that helps the hyphae to penetrate the substrate^15^,^16^,^17^,^18^. We reason that such an orchestrated pressure distribution on the substrate will depend on the mycelial growth pattern and the physical parameters that determine the strength of the fungal hyphae. Interestingly, our recent Q-ACT based studies already demonstrated that physical strength profiles within the mycelia correlate inherently with secondary metabolism^14^ suggesting that pressure profiles generated by fungal colonies are critical determinants of their metabolic state as well as their invasiveness into the substrate. Currently, very little is known about relation between the generation of the pressure by a fungal colony on the substrate and the collective physical behavior of the multicellular system of the fungal colony. The primary reason for this knowledge gap is the absence of an existing methodology to map the pressure profiles exerted by a fungal colony on its substrate. In this work, we introduce an analytical model for computation of the mechanical pressure exerted by an *A. parasiticus* colony.

## Results

### Imaging of the wrinkles in the growth substrate with QACT

**Figure 1:**
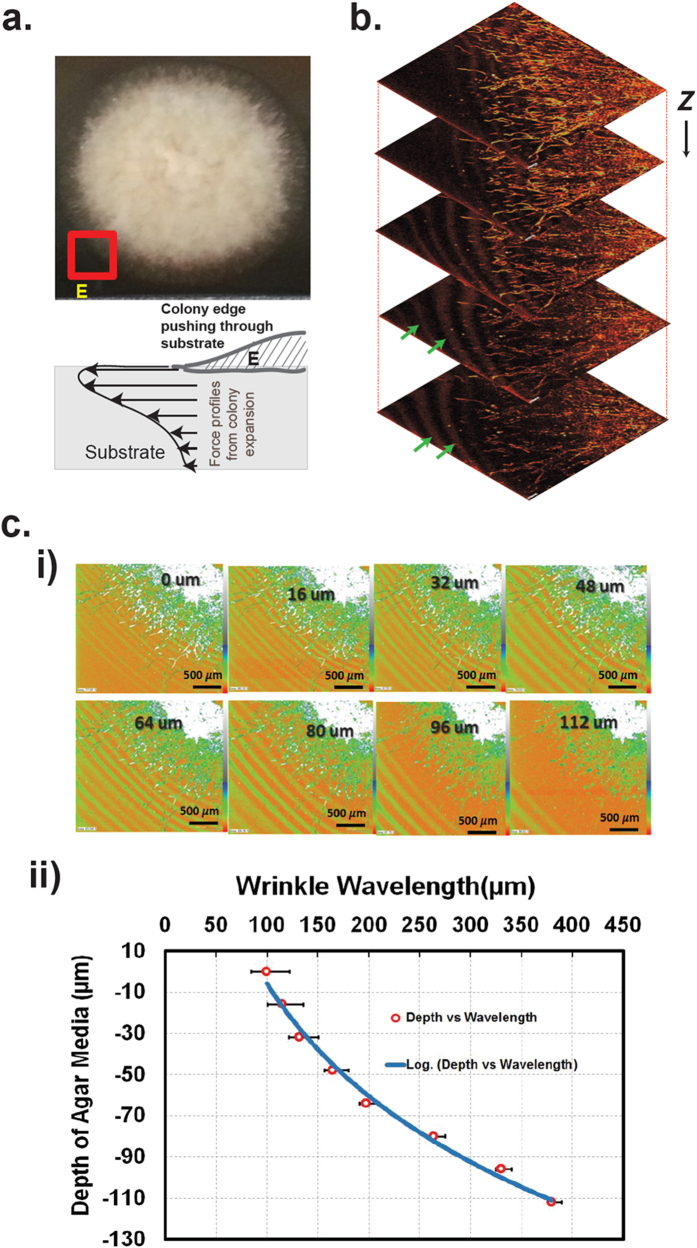
Wrinkle formation within the Aspergillus growth medium. (**a**) *A. parasiticus* grown on solid YES agar growth medium for 2d was studied using Q-ACT. Lower panel illustrates the force profiles exerted on the solid agar substrate from the colony edge within inset E; (**b**) Representative ultrasound micrographs along the depth obtained from Q-ACT at the colony edge within inset E, green arrows denote the wrinkles observed in the substrate due to colony expansion; (**c**) Demonstration of the variation of wrinkle wavelengths along the depths of agar that are 16 μm apart; (**d**) plot of wrinkle wavelength along depth of the substrate

As a first step to establish the mathematical foundation for subsurface pressure profiling, we performed Q-ACT imaging on *A. parasiticus* colonies grown on a nutrient rich growth medium (yeast extract sucrose agar, YES agar). Upon investigation of the tomographs of the substrate below the colony, we surprisingly discovered that the colony growth resulted in the creation of wrinkles in the media, that these were uniform and continuous around and near the edge of the colony (Fig. 1a). Visual assessment of the wrinkles in the tomograph suggested that they were a reflection of the pressure with which the colony pushes the substrate. To compute the pressure that resulted in the wrinkles in the substrate, we next proceeded to understand the relation between the pressure profiles from the colony and the wrinkle patterns created in the substrate. Using Q-ACT we measured the wrinkle wavelengths at different depths (Fig. 1a,b). The measurements revealed that the wrinkle wavelengths were not constant values but varied across the depth (increased with the depth) of the media. Figure 1c shows the mean values of the wrinkle wavelengths across depth obtained from a 2d old *A. parasiticus* colony. We also noted that the wrinkle wavelength (L in *μ*m.) followed a logarithmic pattern across the depth (d in *μ*m.), and could be expressed as a mathematical equation as follows:


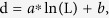


where, parameters ‘a’ and ‘b’ will depend on the fungal species, the growth media and the growth environment. For a 2d colony of *A. parasiticus* grown on YES media, a = ~−79 ± 6.5 and b = ~358 ± 56.

Based on the wavelength profile, we hypothesized that the pressures exerted by the colony were also not uniform across the depth. Hence we proceeded to determine the pressure profiles using a computational analysis based on the fundamental physics of incremental stress.

### Formulation of the relation between substrate wrinkles and the pressure distribution from the fungal colony

**Figure 2:**
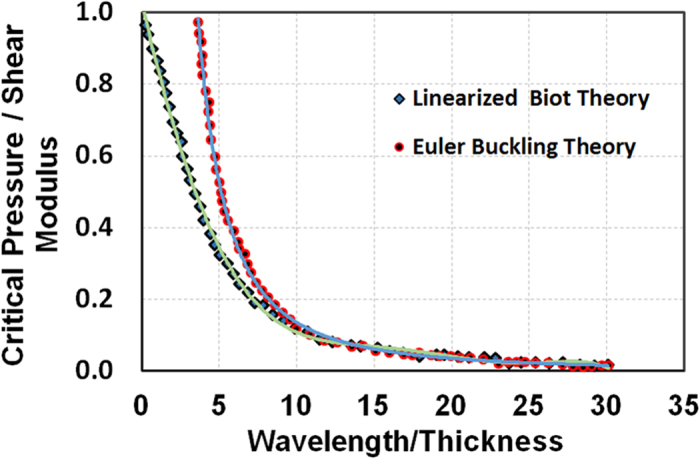
Comparison of Critical pressure/Shear modulus ratio with the Wavelength/Thickness ratio obtained from the Euler buckling theory and the linearized Biot theory. Euler theory predicts that critical pressure goes to infinity when Wavelength/Thickness ratio is less than ~5, whereas, Biot’s theory predicts a finite value at the same range^19^.

Euler buckling theory is conventionally employed in Engineering to predict the critical buckling pressure. According to this theory, Buckling pressure is inherently correlated to the mode of buckling, which represents the number of inflection points in the structure. However, previous studies have indicated that the Euler buckling theory is most effective in predicting the critical buckling pressure when the thickness of the medium is ~5 times less than the wavelength of the instabilities. Below the wavelength/thickness ratio ~5, the Euler theory results infinite critical pressure^19^, as shown in Fig. 2. To avoid such singularity problem, Biot’s incremental stress theory was proposed^19^. Biot’s theory was used extensively in geophysics to study low–amplitude wrinkle formation in the stratified sedimentary rock bed^19^,^20^,^21^,^22^,^23^,^24^. According to this theory, wrinkles originated from the instability caused by compressive load acting parallel to the media^22^,^23^,^25^. A general relation between the wrinkle wavelengths and the critical compressive load was formulated to quantify the pressure required to form the wrinkles in the rock bed. It has been used extensively for the study of folding in isotropic material as well as in anisotropic and viscoelastic media^19^,^20^,^26^,^27^. In this work, since a fungal colony is bounded by its growth medium, we reasoned that the application of incremental stress theory^28^ in viscoelastic media proposed by Biot is not adequate to calculate the critical pressure exerted by a fungal colony.

To address the uniqueness of the wrinkles created by a fungal colony, here we modified the Biot’s formulation and derived the equilibrium equation from the fundamental physics by applying the physics of incremental stress theory, which allowed us to determine the physics of deformations of the agar medium under the initial stress with small incremental perturbations. Incremental stresses at a point is generated due to the displacement and rotation of the continuum body from the reference to deformed configuration^29^. In the conventional linear continuum mechanics incremental stresses are not considered. However, for determination of pressure profiles below the Aspergillus colony, the incremental stress was a critical feature that needed consideration. Based on our visual assessment of creation of wrinkles with fungal growth, we speculated that the when sufficient initial stress builds up on the growth medium due to expansion of the pathogenic colony, wrinkles are developed due to the resistance by the media. As the colony expanded, further pressure applied on the agar media acted as the initial stress, and was further incremented. This incremental pressure was less than the initial pressure that existed in the medium.

**Figure 3:**
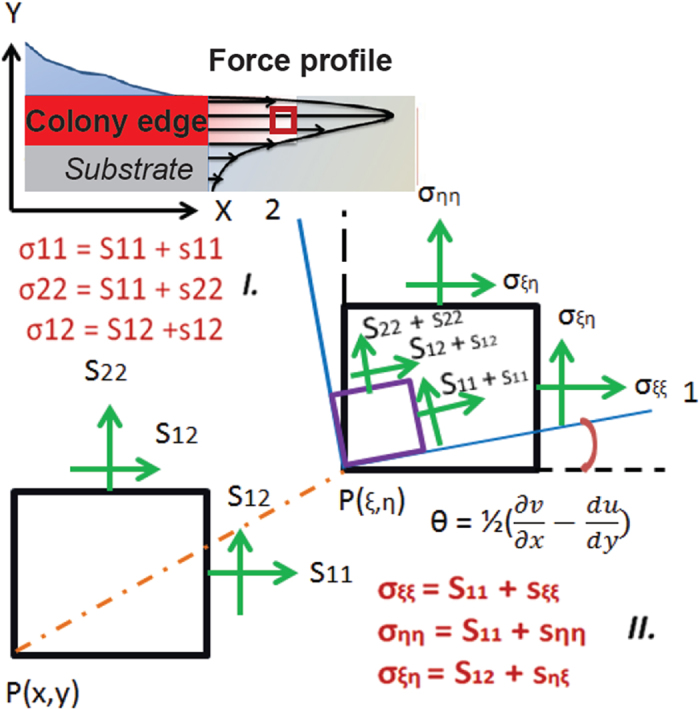
A schematic illustration of our proposed incremental stress model. *Upper panel.* Force profiles resulting from colony edge pushing onto the substrate. Incremental stress condition in the cube within the substrate is shown below. *Lower panel. I*. Representation of initial stresses S11, S12, S22 and the incremental stresses s11, s12, s22. *II.* sξξ, sηη, sηξ are the increment of total stress at the displacement point P(ξ, η) after deformation.

Our proposed analytical model is shown in Fig. 3. The model takes into account the assumption for a fungal colony, that the incremental stress is much smaller than the initial stress as described above. The annotations used to denote the stress fields are described under SI-1. Based on our observations that the colony predominantly expands radially, we considered that the initial stresses acting on the growth medium were only axial stress (S_11_ = P_x_). The vertical pressure (S_22_) and shear stress (S_12_) were neglected based on the assumption that the colony weight was negligibly smaller than the value needed to create a wrinkle in the agar medium. A detailed explanation of the rationale for neglecting the colony weight in our calculations is provided under supplementary mathematical methods (SI-4). Further, in our analysis, vertical pressure is developed due to the accumulation of the fungal biomass on the growth substrate. However, the wrinkles that formed as a result were away from the colony (Fig. 1). Formation of wrinkles were not observed in a 1d old colony but were clearly visible in 2d old colonies, which suggested that a minimum threshold physical strength profile of the colony is needed to exert enough pressure from the colony tips to form such wrinkles. The phenomenon was repeatable in all 12 *A. parasiticus* colonies that were studied. Based on these observations we conducted all the computational analyses for this work with 2d old colonies.

Through rigorous mathematical derivation, applying the incremental stress theory (supplementary mathematical methods SI-2 and SI-3) the linearized equation of equilibrium for incremental displacement could be written as^19^


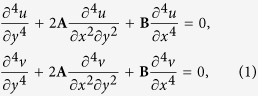


Where, u and v are the displacement of the medium along X & Y direction, respectively. Coefficients **A** and **B** are the function of the initial stresses and the material properties and could be expressed as^19^,


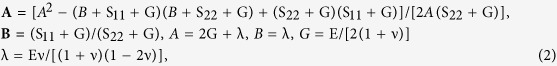


Where, G is modulus of Rigidity, E is Young’s modulus, v is Poisson’s ratio and G and λ are the lame’s constant, respectively. Total displacements are equal to the initial displacement plus the incremental displacement. Incremental boundary stresses which are defined as the differences between the actual boundary forces after deformation and their initial value before deformation were expressed as^30^


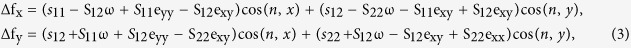


where *S*_11_, *S*_12_ *S*_22_ are the incremental stress components, S_11_,S_22_, and S_12_ are intital stress components, e_xx,_ e_xy,_ and e_yy_ are the strain components,respectively.

Since our Q-ACT revealed that wrinkles are formed in a sinusoidal pattern, we used sinusoidal displacement functions that satisfied the boundary conditions and was assumed to represent the wrinkle formation in the medium. The solution of the equation Eq. (1) therefore could be expressed as^19^


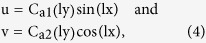


where, l = 2π/L, L is wrinkle wavelength and C_a1_(ly), C_a2_(ly) are the functions of y. We expressed the displacement function as


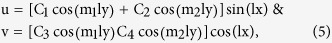


Where, 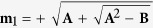 and 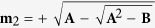.

The displacement functions are the solution of the equilibrium equation and must satisfy the boundary conditions. In order to apply boundary conditions, the displacement functions at the bottom of the agar media should be zero, because the displacements between the interface of the agar and plate are negligible. To impose the boundary conditions at the free surface, we needed to calculate the incremental boundary stresses after the deformation. The initial stress S_11_ = P_x_, acted parallel to the x axis, due to which, the surface is free from the stress. After deformation the free surface deformed as a corrugated sinusoidal surface and incremental stresses (Δfx and Δfy) were generated due to the deformation of the medium. The boundary conditions were applied to represent the physics, u = 0 & v = 0, at Y = 0 and also Δfx = *s*_12_ + P_x_e_xy_ = 0, Δfy = *s*_22_ = 0 at Y = H, traction free surface.

Upon substituting the displacement functions into the boundary conditions and setting S_11_ = Px, S_22_ = 0 and S_12_ = 0 in the incremental stress-strain relationship, 
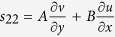
, 
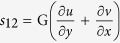
 and
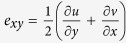
, we obtained four homogenous equations which were written in a matrix form (not shown) and for which, a solution exists if the determinant of the matrix becomes zero. In the matrix the input parameters are wrinkle wavelength, L, height of agar media, H, elastic modulus, E, and the Poisson ratio, ν, respectively. The horizontal pressure, S_11_ = P_x_, was calculated by optimizing the error function when minimum. The horizontal pressure distributions (axial stress) were determined at different wrinkle wavelengths across the depth for all the specimens.

### Determination of pressure profiles on the substrate from the Aspergillus colony

**Figure 4:**
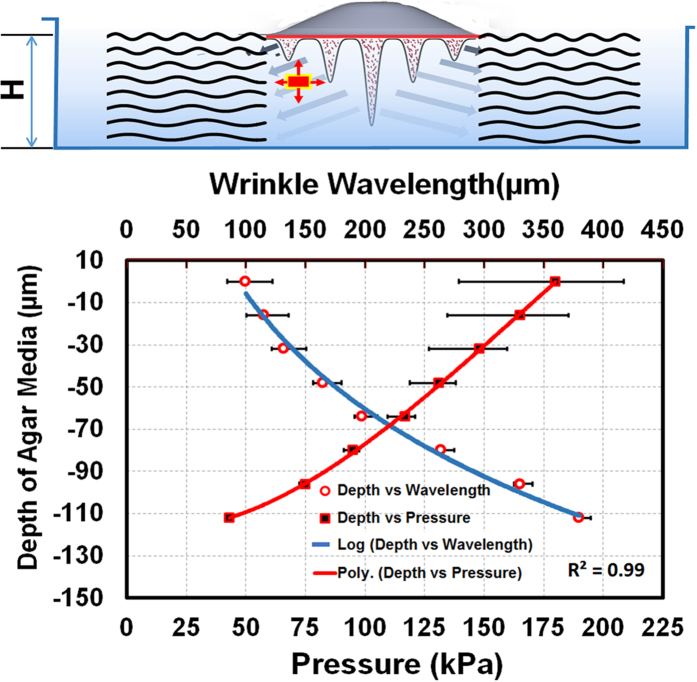
Pressure exerted on the substrate along depth. *Upper panel.* Cartoon describing the wrinkle formation in the substrate as a result of the Aspergillus expansion. *Lower panel.* Pressure values computed along depth. Mean values of the wrinkle wavelengths across different depths of the media are also shown alongside pressure values along the depth of the substrate.

To determine the pressure from the *A. parasiticus* colony that created the observed wrinkles in Fig. 1, we incorporated in our calculations the following values: depth of the agar media, H = 2.14 ± 0.08 mm, Elastic Modulus, E, of the Agar media = 700 ± 21 kPa, that was determined from the wave velocity in the medium^14^,^31^, and Poisson ratio, 0.49. Figure 4 depicts the mean value of the pressure distribution across the depth obtained from 12 specimens. Our results show that the pressure decreased with depth following a third order polynomial function. This can be explained by the fact that wavelength increased with depth and hence reduced pressure was required to wrinkle the media. Our results are also in line with our previous observations^14^ that the strength of the fungus colony decreases with the depth, possibly due to the significant decrease in number of active hyphae compared to the surface.

**Figure 5:**
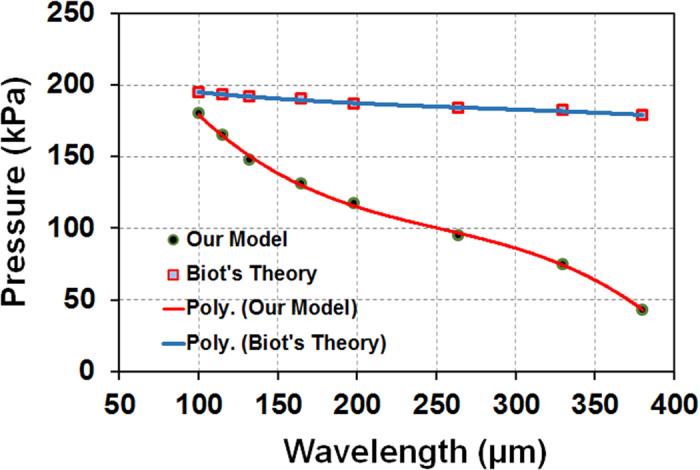
Comparison of pressure values for different wavelengths calculated from our incremental stress model and Biot’s theory. Biot model predicts almost contact pressure for different wrinkle wavelengths, which is a significant divergence from the reality. On the contrary, our analytical model was able to describe the variations in pressure with the variation at different wavelengths.

Finally, to demonstrate that our model is more realistic than the Biot’s model to show the variations in pressure with the varying wrinkle wavelengths (with substrate depth), we have conducted a comparison of the pressure vs. wavelength relation rendered from Biot’s model^19^ and our model for a single isolated layer with same wavelength/thickness ratio (~5). The results are shown in Fig. 5. Our results show that although Biot’s model successfully avoided the drawbacks of Euler’s predictions at wavelength/thickness less than ~5 (in which the critical pressure diverges towards the infinite^19^), it still predicted almost constant pressure with varying wrinkle wavelengths. Our model on the contrary successfully depicted the variations of pressure that resulted in variations of wavelength in the substrate. There was a good agreement, as we expected, between Biot’s and our model when the wrinkle wavelengths are smaller (<100 *μ*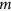) with H = 2.4 mm. However they constantly diverged with increasing wrinkle wavelengths, with pressure values ranging within ~100–180 KPa. This phenomenon can be explained by the fact that smaller wavelength at the top of agar media neglects the effects from the boundary constraints and reflects same physics as a single isolated layer described by Biot^22^,^23^,^25^. As the wavelength increases across the depth of media the boundary constraints dominate in the critical pressure calculation and thus diverge. Hence, we report our model as a more generalized incremental stress model with boundary effect.

## Discussion

Here we demonstrate for the first time, the feasibility of development of analytical models to study pressure profiles exerted by a fungal colony on its substrate, which in turn, depend on the 3D physical property profiles of the colony. To establish this initial mathematical infrastructure we computed the mechanical pressure exerted on a solid agar growth medium, from a colony of the plant pathogen, *A. parasiticus,* by measuring the wavelengths of the wrinkles that the fungus generates in the medium during colony expansion and establishing the generalized equilibrium equations of incremental stresses from these measurements. The creation of wrinkles in solid growth medium (a most realistic model of the substrates on which fungi grow in nature) was not previously reported and is also a novel finding in this study. Further, the novel analytical technique proposed here could successfully determine the critical pressure for low amplitude/ thickness ratio with constrained boundary where Euler buckling theory and Biot’s theory could not be used to predict the critical pressure accurately. We also emphasize here that developing such a mathematical infrastructure was possible only due to the 3D wavelength measurements generated by Q-ACT. In our previous studies^14^ we had established the technique as the most non-invasive method suited for 3D imaging of fungal colonies compared to existing state of the art quantitative ultrasonic methods. Here we demonstrate here that Q-ACT can also be used to obtain subsurface information across the depth of a fungal substrate.

Our results suggest that the changes in colony morphologies with growth that were observed in our previous work^14^ could be deterministic in the formation of the uniform and continuous wrinkles around and near the edge of the colony. Our current work is focused on performing a series of time-dependent experiments using an array of different media and *A. parasiticus* mutants to provide quantitative comparisons of ‘threshold morphologies’ that are needed to initiate such wrinkles. These time-dependent experiments will be key in studying the role of different morphological parameters on fungal pressures exerted on the substrate.

Finally, it should also be noted that the pressure profiles exerted from a fungal colonies are a function of the material property of the substrate. Hence, same wavelength could result in different pressure magnitudes. Pressure profile with higher magnitude in a substrate with similar strength is the result of higher strength of the colony and hence implicates greater severity of fungal invasion. Our future studies will use the mathematical foundation developed in this work to model pressure profiles generated by fungal pathogens in plant and animal tissues.

## Additional Information

**How to cite this article**: Patra, S. *et al.* Subsurface pressure profiling: a novel mathematical paradigm for computing colony pressures on substrate during fungal infections. *Sci. Rep.* **5**, 12928; doi: 10.1038/srep12928 (2015).

## Supplementary Material

Supplementary Information
