# Supplementary material for: Subsurface pressure profiling: a novel mathematical paradigm for computing colony pressures on substrate during fungal infections
Source: Sci Rep. 2015 Aug 11;5:12928. doi: 10.1038/srep12928 (PMC4531784; doi:10.1038/srep12928)
Supplement: Supplementary Information [file srep12928-s1.pdf]

Subsurface pressure profiling: a novel mathematical paradigm for computing colony pressures on substrate during fungal infections

Subir Patra[1,†], Sourav Banerjee[1*,†], Gabriel Terejanu[2], Anindya Chanda[3*]

[1]Integrated Material Assessment and Predictive Simulation Laboratory, Department of Mechanical Engineering, University of South Carolina, Columbia, SC 29208, USA

[2]Department of Computer Science and Engineering, University of South Carolina, Columbia, SC 29208, USA

[3]Department of Environmental Health Sciences, Arnold School of Public Health, University of South Carolina, Columbia, SC 29208, USA

[†]These authors contributed equally to the manuscript.

*Corresponding Authors: banerjes@cec.sc.edu ; achanda@mailbox.sc.edu

Supplementary Mathematical Methods

SI- 1: Annotations for describing stress field

For our mathematical derivations, we followed the standard annotations of Solid Mechanics. The x, y and z directions in 3D coordinate system are designated here as 1, 2 and 3, respectively. S_{ij} (black arrow in the graph, SI Fig. 1) denotes the stress on a surface which is perpendicular to the direction *i* and the stress acting along the direction *j*. We point out here that *i*, *j* can take any values between 1,2 and 3, which are synonymous to x, y and z directions. Hence, in a perturbed zone, S_{11} and S_{22} represents the normal stresses acting on a square element along x, y direction, respectively, and S_{12} is the shear stress.

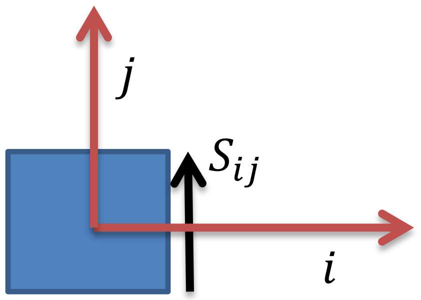

SI Fig. 1: Showing index convention of stress

Further, when an element is displaced form one point to another point, increment stresses are developed on the deformed element due to rotation and displacement of element. The rotation of the initial stress field during displacement from one point to another will result in a new stress field of the deformed element that can be described in a rotated coordinate system by the following stresses, σ_{11}, σ_{22} and σ_{12}, respectively. Here,

$$\sigma_{11} = \mathrm{S}_{11} + s_{11}, \sigma_{22} = \mathrm{S}_{22} + s_{22} \text{ and } \sigma_{12} = \mathrm{S}_{12} + s_{12},$$

where,
s_{11}, s_{22} and s_{12} are incremental stresses developed during displacement of an element from one point to another. These incremental stresses were used to develop equation of equilibrium, which by using linear stress- stress equation, eventually converted into a system of differential equations with displacement as dependent variables,.

SI- 2: Derivation of equation 1[19]

To derive the equations for this work the derivation steps performed as described previously[19]. In Eulerian representation of coordinate system (deformed coordinate system) the equation of equilibrium of a material element (square shape after deformation as illustrated in SI Fig. 2) is written as

$$\frac{\partial \sigma_{\xi\xi}}{\partial \xi} + \frac{\partial \sigma_{\xi\eta}}{\partial \eta} + F_\xi = 0$$

$$\frac{\partial \sigma_{\xi\eta}}{\partial \xi} + \frac{\partial \sigma_{\eta\eta}}{\partial \eta} + F_\eta = 0$$

$$\sigma_{\xi\eta} = \sigma_{\eta\xi}$$

We first transformed this equation to the undeformed coordinate system.

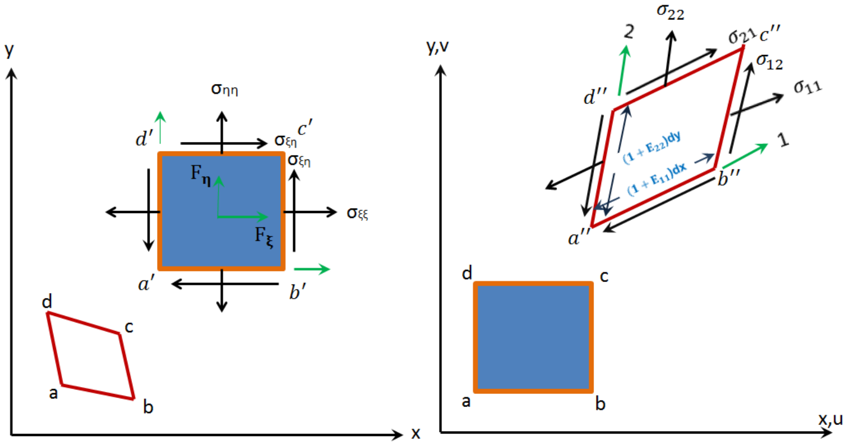

SI Fig. 2: A material point under deformation

The following geometrically nonlinear strain displacement relation (also illustrated in SI Fig. 3) was used to address the large deformation condition for the fungal growth media:

$$\varepsilon_{xx} = \frac{\partial u}{\partial x} + \frac{1}{2}\left[\left(\frac{\partial u}{\partial x}\right)^2 + \left(\frac{\partial v}{\partial x}\right)^2\right]$$

$$\varepsilon_{yy} = \frac{\partial v}{\partial y} + \frac{1}{2}\left[\left(\frac{\partial u}{\partial y}\right)^2 + \left(\frac{\partial v}{\partial y}\right)^2\right]$$

$$\varepsilon_{xy} = \frac{1}{2}\left[\frac{\partial u}{\partial y} + \frac{\partial v}{\partial x} + \frac{\partial^2 u}{\partial x \partial y} + \frac{\partial^2 v}{\partial x \partial y}\right]$$

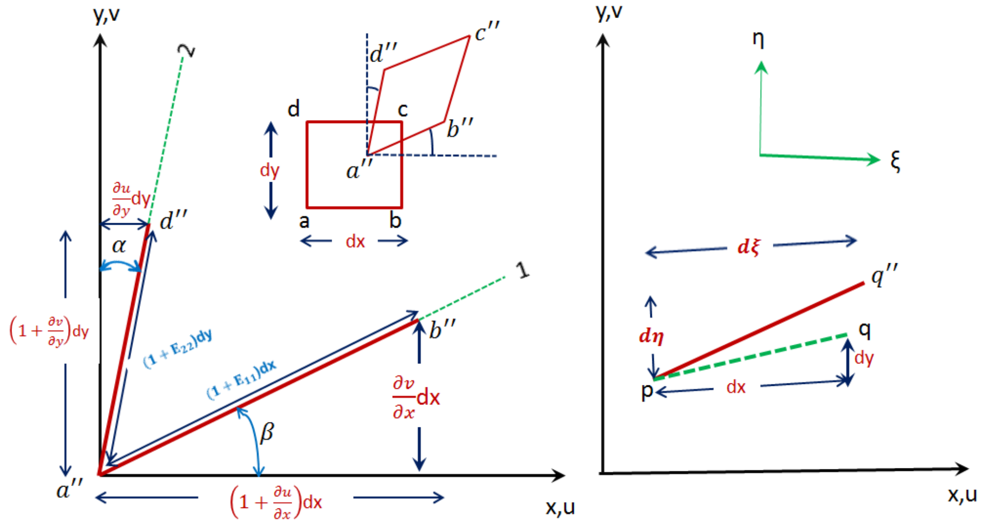

SI Fig. 3: Deformation (Extension and rotation of element edges)

We substituted the nonlinear strain displacement relation into the governing equation transformed to the undeformed coordinate system and obtained the following differential equations after several mathematical steps (details of the mathematical derivation not shown):

$$\frac{\partial}{\partial x}\left[\sigma_{\xi\xi}\left(1+\frac{\partial v}{\partial y}\right)-\sigma_{\eta\xi}\left(\frac{\partial u}{\partial y}\right)\right]+\frac{\partial}{\partial y}\left[\sigma_{\eta\xi}\left(1+\frac{\partial u}{\partial x}\right)-\sigma_{\xi\xi}\left(\frac{\partial v}{\partial x}\right)\right]+\sigma_{\xi\xi}\left\{\frac{\partial^2 v}{\partial x\partial y}-\frac{\partial}{\partial x}\left(1+\frac{\partial v}{\partial y})\right)\right\}$$
$$+\sigma_{\eta\xi}\left\{\frac{\partial^2 u}{\partial x\partial y}-\frac{\partial}{\partial y}\left(1+\frac{\partial u}{\partial x})\right)\right\}+DF_{\xi}=0$$

$$\frac{\partial}{\partial x}\left[\sigma_{\xi\eta}\left(1+\frac{\partial v}{\partial y}\right)-\sigma_{\eta\eta}\left(\frac{\partial u}{\partial y}\right)\right]+\frac{\partial}{\partial y}\left[\sigma_{\eta\eta}\left(1+\frac{\partial u}{\partial x}\right)-\sigma_{\xi\eta}\left(\frac{\partial v}{\partial x}\right)\right]+\sigma_{\eta\eta}\left\{\frac{\partial^2 u}{\partial x\partial y}-\frac{\partial}{\partial y}\left(1+\frac{\partial u}{\partial x})\right)\right\}$$
$$+\sigma_{\eta\xi}\left\{\frac{\partial^2 u}{\partial x\partial y}-\frac{\partial}{\partial x}\left(1+\frac{\partial v}{\partial y})\right)\right\}+DF_{\eta}=0$$

These equations upon further simplification rendered the following:

$$\frac{\partial}{\partial x}\left[\sigma_{\xi\xi}\left(1+\frac{\partial v}{\partial y}\right)-\sigma_{\eta\xi}\left(\frac{\partial u}{\partial y}\right)\right]+\frac{\partial}{\partial y}\left[\sigma_{\eta\xi}\left(1+\frac{\partial u}{\partial x}\right)-\sigma_{\xi\xi}\left(\frac{\partial v}{\partial x}\right)\right]+DF_{\xi}=0$$

$$\frac{\partial}{\partial x}\left[\sigma_{\xi\eta}\left(1+\frac{\partial v}{\partial y}\right)-\sigma_{\eta\eta}\left(\frac{\partial u}{\partial y}\right)\right]+\frac{\partial}{\partial y}\left[\sigma_{\eta\eta}\left(1+\frac{\partial u}{\partial x}\right)-\sigma_{\xi\eta}\left(\frac{\partial v}{\partial x}\right)\right]+DF_{\eta}=0$$

Next we incorporated the concept of initial and incremental stress state. Following to the previous equations we re-wrote the equilibrium equation (SI Fig. 4) in deformed 1, 2 coordinate system as follows.

$$\frac{\partial}{\partial x}\left[\sigma_{11}\left(\frac{1+E_{22}}{1+E_{11}}\right)\left(1+\frac{\partial u}{\partial x}\right)+\sigma_{12}\left(\frac{\partial u}{\partial y}\right)\right]+\frac{\partial}{\partial y}\left[\sigma_{22}\left(\frac{1+E_{11}}{1+E_{22}}\right)\left(\frac{\partial u}{\partial y}\right)+\sigma_{21}\left(1+\frac{\partial u}{\partial x}\right)\right]+DF_{\xi}=0$$

$$\frac{\partial}{\partial x}\left[\sigma_{11}\left(\frac{1+E_{22}}{1+E_{11}}\right)\left(\frac{\partial v}{\partial x}\right)+\sigma_{12}\left(1+\frac{\partial v}{\partial y}\right)\right]+\frac{\partial}{\partial y}\left[\sigma_{22}\left(\frac{1+E_{11}}{1+E_{22}}\right)\left(1+\frac{\partial v}{\partial y}\right)+\sigma_{21}\left(\frac{\partial v}{\partial x}\right)\right]+DF_{\boldsymbol{\eta}}=0$$

$$\boldsymbol{\sigma_{12}=\sigma_{21}}$$

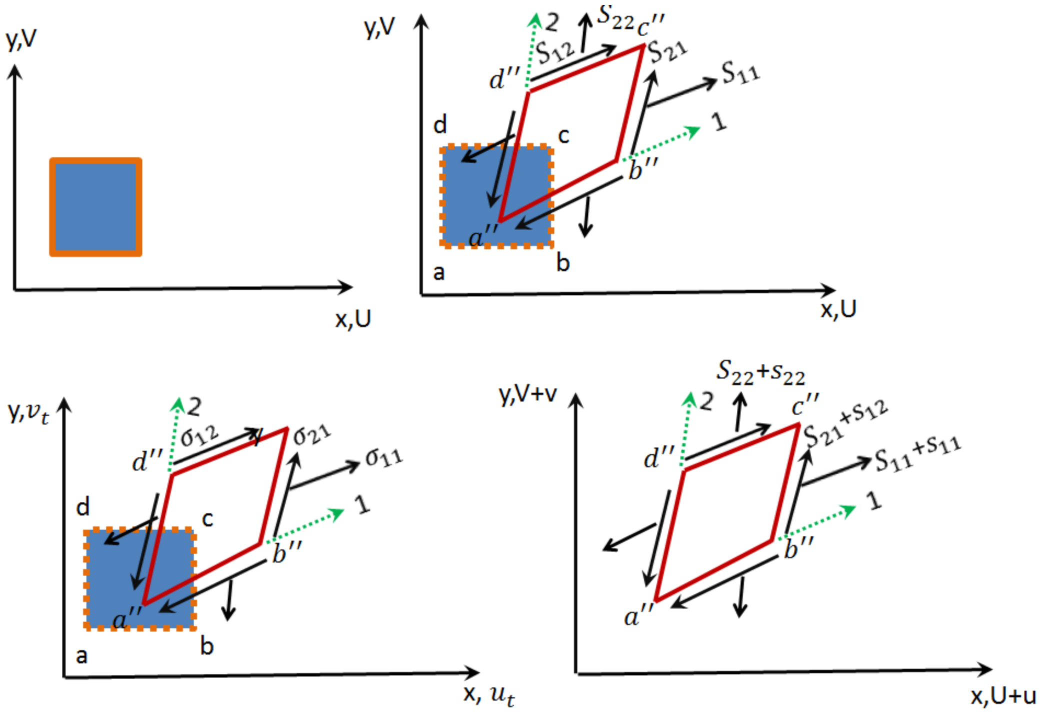

SI Fig. 4: Incremental and Initial Stress state

In contrast to linear elastic theory, the stress state here (in deformed 1, 2 coordinate system) was the summation of the initial stress and the incremental stress (as shown in Figure 5) because it is defined as the rotation of the material point that will cause an additional incremental stress in the deformed coordinate system. This rendered the following after several mathematical steps (not shown here):

$$S_{11}\frac{\partial^2 v}{\partial x\partial y}+\frac{\partial s_{11}}{\partial x}+S_{22}\frac{\partial^2 u}{\partial y^2}+2S_{12}\frac{\partial^2 u}{\partial x\partial y}+\frac{\partial s_{21}}{\partial y}=0$$

$$S_{11}\frac{\partial^2 v}{\partial x^2}+\frac{\partial s_{12}}{\partial x}+S_{22}\frac{\partial^2 u}{\partial x\partial y}+2S_{12}\frac{\partial^2 v}{\partial x\partial y}+\frac{\partial s_{22}}{\partial y}=0$$

Substituting stress displacement relation we got

$$A\frac{\partial^2 u}{\partial x^2}+(B+S_{11}+G)\frac{\partial^2 v}{\partial x\partial y}+(G+S_{22})\frac{\partial^2 u}{\partial y^2}=0$$

$$A\frac{\partial^2 v}{\partial y^2}+(B+S_{22}+G)\frac{\partial^2 u}{\partial x\partial y}+(G+S_{11})\frac{\partial^2 v}{\partial x^2}=0$$

Where, *A*=2G+λ, *B*=λ, G=E/ [2(1+ v)]

λ=Ev /[(1+ v)(1-2v)],

Where, G is modulus of Rigidity, E is Young's modulus, v is Poisson's ratio and G and λ are the lame’s constant, respectively.

Please note that the above equations have both displacement variables in both equations. Algebraic operation on these rendered the following two decoupled equations (**Equation 1**) :

$\frac{\partial^4 u}{\partial y^4}$+2**A** $\frac{\partial^4 u}{\partial x^2\partial y^2}$+**B** $\frac{\partial^4 u}{\partial x^4}$=0,

$\frac{\partial^4 v}{\partial y^4}$+2**A** $\frac{\partial^4 v}{\partial x^2\partial y^2}$+**B** $\frac{\partial^4 v}{\partial x^4}$=0,

where, u and v are the displacement of the medium along X & Y direction, respectively. Coefficients **A** and **B** are the function of the initial stresses and the material properties and could be expressed as[19],

A= [A^2-(*B*+S_{11}+G) (*B*+S_{22}+G) + (S_{22}+G) (S_{11}+G)]/ [2*A* (S_{22}+G)],

B= (S_{11}+G)/ (S_{22}+G),

SI- 3. Boundary Condition

We briefly formulate here the equations for boundary conditions (also illustrated in SI Fig. 4). The step-by-step mathematical process shown here will be self-explanatory from a solid mechanics point of view.

.

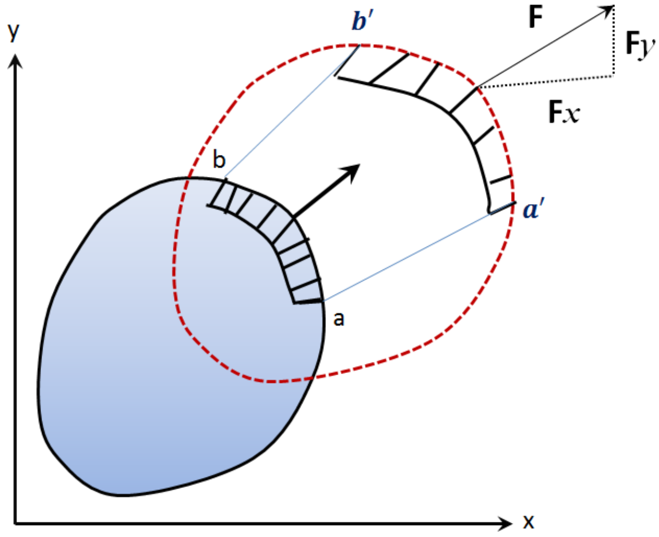

Boundary force F acting on deformed boundary $a'b'$
ab is finite portion of original boundary

SI Fig. 5: Equations for boundary condition

$$F_x = \int_{a'}^{b'} F_x \, ds' = \int_{a'}^{b'} (\sigma_{\xi\xi} d\boldsymbol{\eta} - \sigma_{\xi\eta} \partial\boldsymbol{\xi})$$

$$F_y = \int_{a'}^{b'} F_y \, ds' = \int_{a'}^{b'} (\sigma_{\xi\eta} d\boldsymbol{\eta} - \sigma_{\eta\eta} \partial\boldsymbol{\xi})$$

$$F_x = \int_a^b \left[\sigma_{\xi\xi} \frac{\partial v}{\partial x} - \sigma_{\xi\eta}\left(1 + \frac{\partial u}{\partial x}\right)\right] dx + \int_a^b \left[\sigma_{\xi\xi}\left(1 + \frac{\partial v}{\partial y}\right) - \sigma_{\xi\eta} \frac{\partial u}{\partial y}\right] dy$$

$$F_y = \int_a^b \left[\sigma_{\xi\eta} \frac{\partial v}{\partial x} - \sigma_{\eta\eta}\left(1 + \frac{\partial u}{\partial x}\right)\right] dx + \int_a^b \left[\sigma_{\xi\eta}\left(1 + \frac{\partial v}{\partial y}\right) - \sigma_{\eta\eta} \frac{\partial u}{\partial y}\right] dy$$

$$dF_x = \left(-\sigma_{\xi\eta} + \sigma_{\xi\xi} \frac{\partial v}{\partial x} - \sigma_{\xi\eta} \frac{\partial u}{\partial x}\right) dx + \left(\sigma_{\xi\xi} + \sigma_{\xi\xi} \frac{\partial v}{\partial y} - \sigma_{\xi\eta} \frac{\partial u}{\partial y}\right) dy$$

$$dF_y = \left(-\sigma_{\eta\eta} + \sigma_{\xi\eta} \frac{\partial v}{\partial x} - \sigma_{\eta\eta} \frac{\partial u}{\partial x}\right) dx + \left(\sigma_{\xi\eta} + \sigma_{\xi\eta} \frac{\partial v}{\partial y} - \sigma_{\eta\eta} \frac{\partial u}{\partial y}\right) dy$$

$$dF_x = -(S_{11} + s_{11} - S_{22}w - S_{11}\varepsilon_{xy} + S_{12}\varepsilon_{xx})dx + (S_{11} + s_{11} - S_{22}w + S_{11}\varepsilon_{xy} - S_{12}\varepsilon_{xx})dy$$

$$dF_y = -(S_{22} + s_{22} + S_{12}w - S_{12}\varepsilon_{xy} + S_{22}\varepsilon_{xx})dx + (S_{12} + s_{12} + S_{11}w + S_{12}\varepsilon_{yy} - S_{22}\varepsilon_{xy})dy$$

$$w = \frac{1}{2}\left(\frac{\partial v}{\partial x} - \frac{\partial u}{\partial y}\right) ; -dx = ds cos(n,y); dy = ds cos(n,x) ; ds = \sqrt{dx^2 + dy^2}$$

Force components can be written as

$$f_x = \frac{dF_x}{ds} \quad ; \qquad f_y = \frac{dF_y}{ds}$$

$$f_x == -(S_{11} + s_{11} - S_{22}w - S_{11}\varepsilon_{xy} + S_{12}\varepsilon_{xx})cos(n,x) + (S_{11} + s_{11} - S_{22}w + S_{11}\varepsilon_{xy} - S_{12}\varepsilon_{xx})cos(n,y)$$

$$f_y = -(S_{22} + s_{22} + S_{12}w - S_{12}\varepsilon_{xy} + S_{22}\varepsilon_{xx})cos(n,x) + (S_{12} + s_{12} + S_{11}w + S_{12}\varepsilon_{yy} - S_{22}\varepsilon_{xy})cos(n,y)$$

This rendered **Equation 3** as follows:

$$\Delta f_x == -(s_{11} - S_{22}w - S_{11}\varepsilon_{xy} + S_{12}\varepsilon_{xx})cos(n,x) + (s_{11} - S_{22}w + S_{11}\varepsilon_{xy} - S_{12}\varepsilon_{xx})cos(n,y)$$

$$\Delta f_y = -(s_{22} + S_{12}w - S_{12}\varepsilon_{xy} + S_{22}\varepsilon_{xx})cos(n,x) + (s_{12} + S_{11}w + S_{12}\varepsilon_{yy} - S_{22}\varepsilon_{xy})cos(n,y)$$

Application of the boundary condition is shown in SI Fig. 6

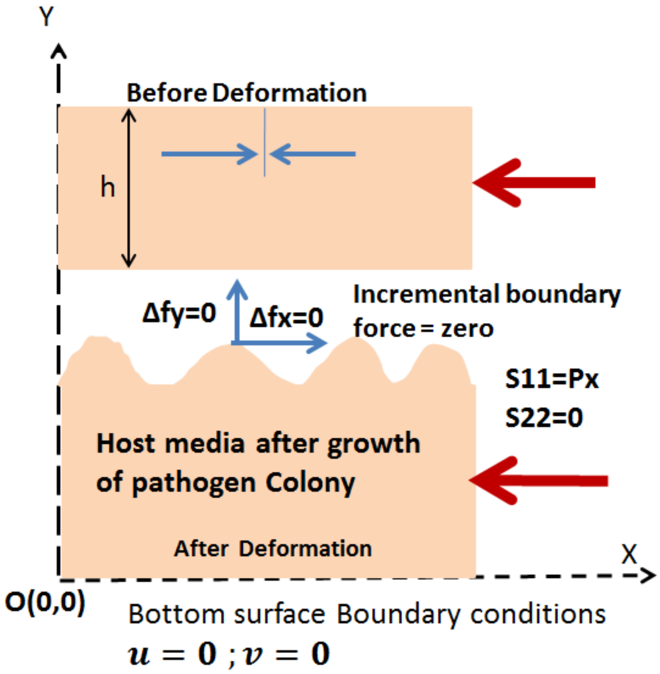

SI Fig. 6: Traction free and displacement boundary conditions

SI- 4. Rationale for neglecting vertical pressure and shear stress

The zero vertical pressure and zero shear stress are called traction free boundary conditions in solid mechanics. For our calculations we neglected any effects from shear stresses because there is no applied shear traction on the surface of the agar. The rationale for neglecting the fungal biomass (source of vertical pressure) is discussed below.

Fungal biomass accumulated on the agar media acts as a circular patch loading on the surface. The vertical stress distribution zone in the agar media was be determined by Boussinesq isobar and is illustrated in SI Fig.7.

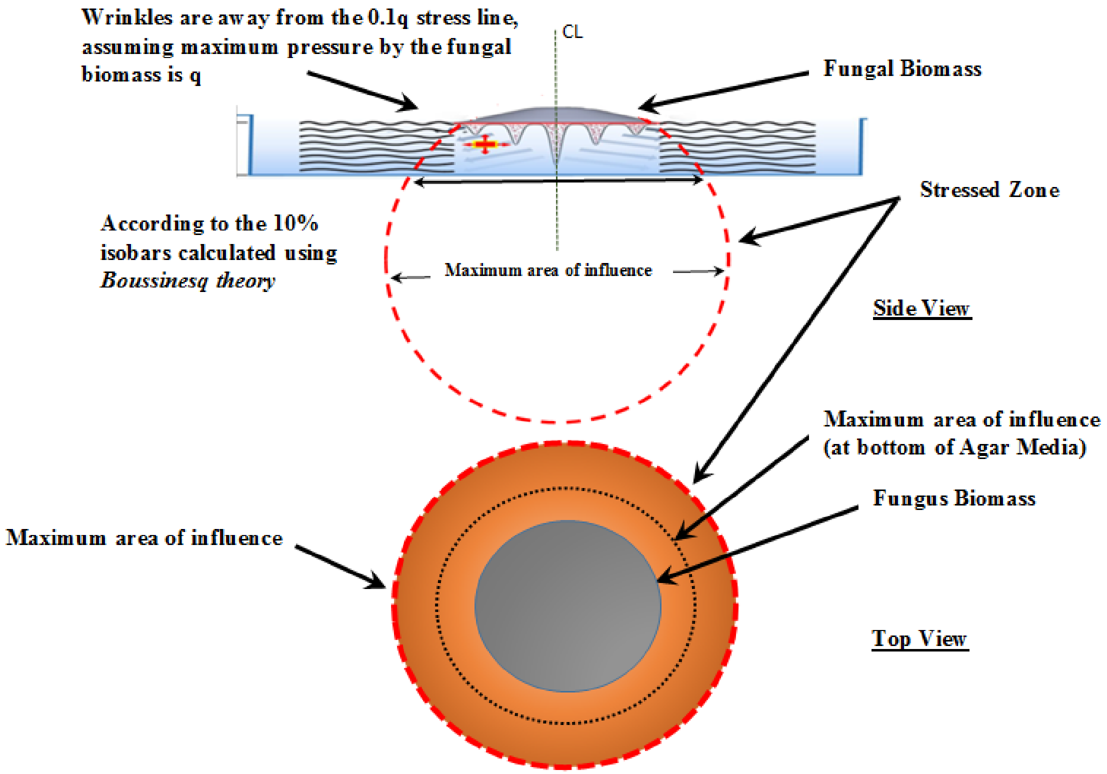

SI Fig. 7. Boussinesq isobar for circular patch loading displaying 10% isobar profile (red dashed line)

As will be evident from this figure, even though the area of influence of the vertical stress (as evident from SI Fig. 7) is greatest at the bottom and lowest at the surface, our QACT images in Fig.1c revealed that wrinkles at the surface were closest to edge of the colony. This distance increased with depth of the medium until at the very bottom of the plate, when wrinkles were formed farthest away from the edge (SI Fig. 8). This strongly suggested the negligible influence of the vertical stress on the observed wrinkle formation. Moreover, we also observed that most of the biomass generated by the colony was accumulated close to centerline symmetrically, rendering a conical shape of the colony. We reasoned that this requires longer anchor length symmetrically close to centerline for the stability of the colony; hence vertical stresses are highest near to the centerline and decrease monotonically with distance from colony edge.

Hence it was very less likely that vertical stresses (colony weight) had any significant role in the observed wrinkle formation at the edge.

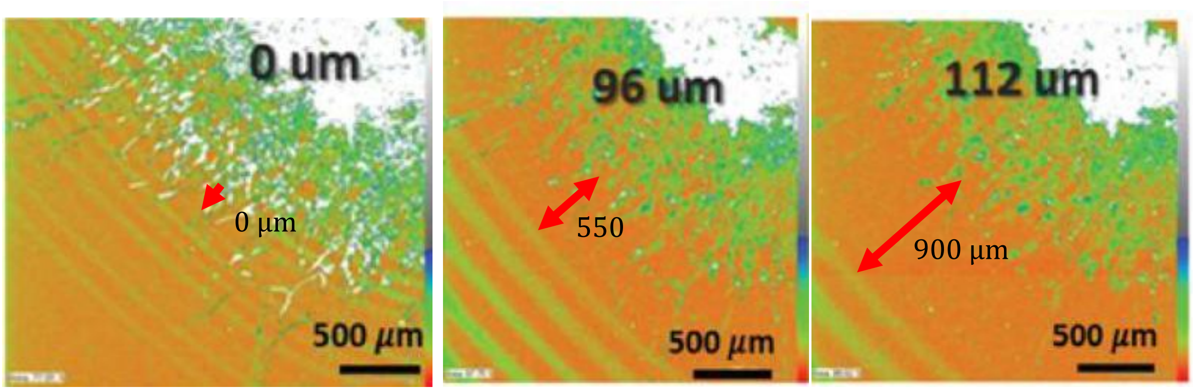

SI Fig. 8. Distance of wrinkle formation region from fungus colony end at different depth of agar media. (QACT images)
